# Supplementary figures and images for: The nuclear poly(A)-binding protein Pab2/PABPN1 promotes heterochromatin assembly through the formation of Pab2 nuclear condensates
Source: PLoS Genet. 2025 Mar 31;21(3):e1011647. doi: 10.1371/journal.pgen.1011647 (PMC12002642; doi:10.1371/journal.pgen.1011647)

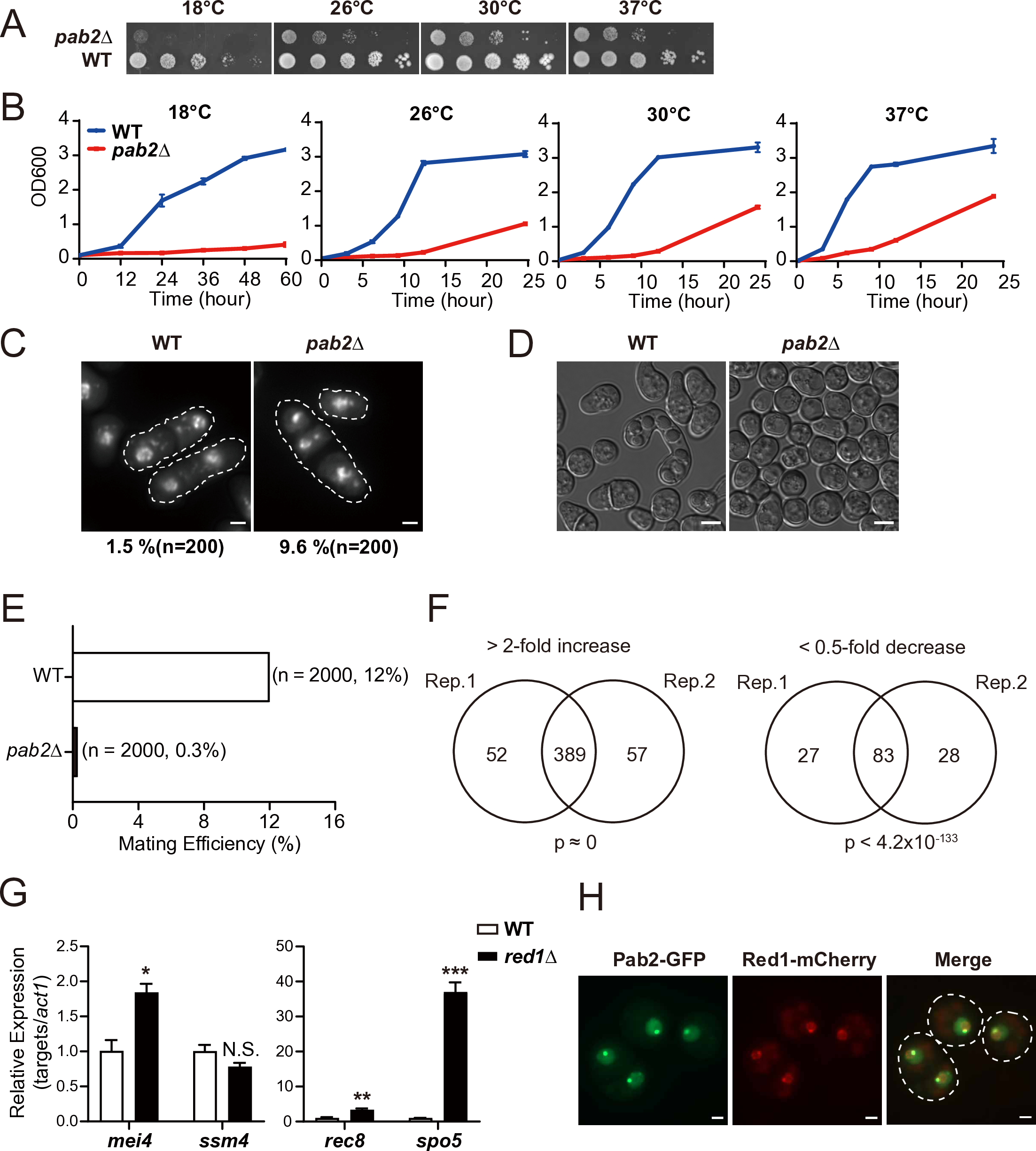

Supplement: S1 Fig — (A) Growth assay of wild-type (WT) and pab2Δ cells. Ten-fold serial dilutions were spotted onto complete medium plates and incubated at the indicated temperatures. (B) WT and pab2Δ cells were grown in complete liquid media, and OD600 was measured at the indicated temperatures. Data represent mean ± SD of three independent experiments. (C) Microscopic analyses of DAPI-stained WT and pab2Δ cells. Scale bars, 2 μm. The percentage of cells with abnormal DNA and the number of cells counted are shown below each panel. (D) DIC images of WT and pab2Δ cells were taken after sporulation induction. Scale bars, 2 μm. (E) Homothallic WT and pab2Δ cells were subjected to a nitrogen-depleted condition, and mating efficiency was assessed by counting 2000 cells under a microscope. Scale bars, 2 μm. (F) Differentially expressed genes (DEGs) in pab2∆ (two biological replicates) are shown in Venn diagrams. The left diagram shows genes with > 2-fold increased expression, and the right diagram shows genes with < 0.5-fold decreased expression. For each comparison, the same set of 5,272 genes was examined. The statistical significance (p-value) of the overlap between each of the two groups using a hypergeometric test is shown under the diagrams. (G) Putative meiotic gene expression in WT and red1Δ. RT-qPCR analysis of four inferred meiotic genes in WT and red1Δ cells. The four putative meiotic mRNAs (mei4, ssm4, rec8, and spo5) were normalized to act1 mRNA to determine their relative expression levels. Data are presented as mean ± SD from three independent experiments. Statistical significance was determined using a two-tailed unpaired t-test (*p < 0.05, **p < 0.01, and ***p < 0.005; N.S.: not significant). (H) The localization of Pab2-GFP and Red1-mCherry during vegetative growth was examined by fluorescent microscopy. Scale bars, 2 μm. (TIF) [file pgen.1011647.s001.tif]

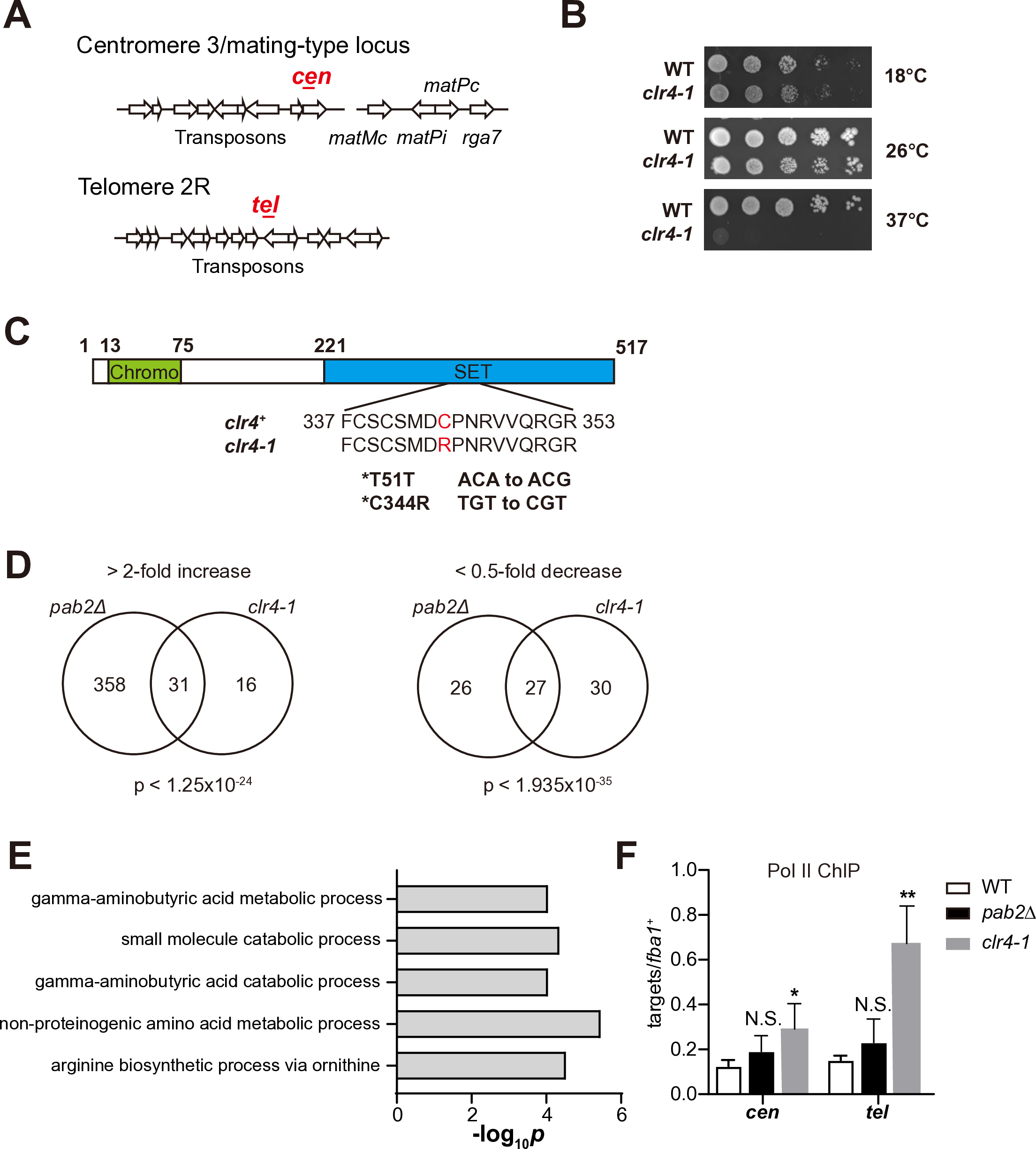

Supplement: S2 Fig — (A) Schematic representation of the amplicons used in this study. The position of cen and tel amplicons, located within the pericentromeric heterochromatin of centromere 3 and the right end of chromosome 2 (Telomere 2R), respectively, is depicted. (B) Growth assay of wild-type (WT) and clr4–1 cells. Ten-fold serial dilutions were spotted onto complete medium plates and incubated at the indicated temperatures. (C) Schematic representation of the Clr4 protein and the mutations found in clr4–1. Clr4 has a chromodomain (Chromo) and a SET domain. The clr4–1 allele contains two base substitutions (A153G and T1030C), and only the T1030C substitution resulted in the C334R substitution in the SET domain. (D) Venn diagrams show differentially expressed genes (DEGs) (left: > 2-fold increased expression; right: < 0.5-fold decreased expression) in pab2∆ and clr4–1 cells. The statistical significance (p-value) of the overlap between each of the two groups using a hypergeometric test is shown under the diagrams. (E) GO analysis of the downregulated genes in pab2Δ and clr4–1 cells. The Y-axis represents the GO annotations (e.g., gamma-aminobutyric acid metabolic process). The X-axis represents the significance of the enriched annotations (-log10p). (F) ChIP-qPCR analysis of RNA polymerase II (Pol II) occupancy at centromere 3 (cen) and telomere 2R (tel) in WT, pab2∆, and clr4–1. Data are presented as mean ± SD from three independent experiments. Statistical significance was determined using a one-way ANOVA followed by Dunnett’s multiple comparison test, with WT as the reference sample (*p < 0.05 and **p < 0.01; N.S.: not significant). (TIF) [file pgen.1011647.s002.tif]

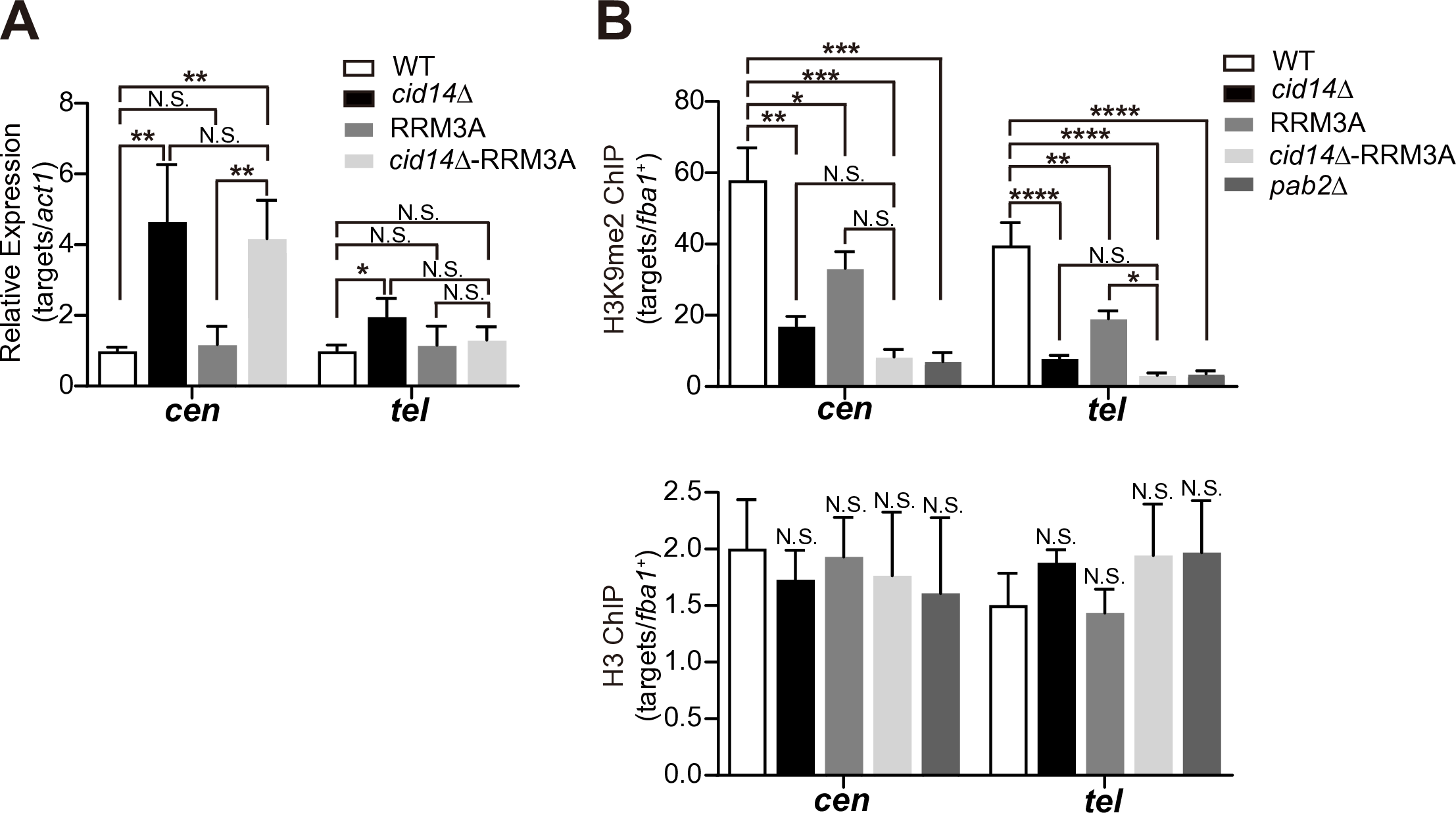

Supplement: S3 Fig — (A) RT-qPCR analysis of centromeric and telomeric transcripts in WT, cid14Δ, pab2RRM3A, and cid14Δ-RRM3A strains. Centromeric (cen) and telomeric (tel) transcripts were normalized to act1 mRNA to determine their relative expression levels. Data are presented as mean ± SD from three independent experiments. Statistical significance was determined using a one-way ANOVA followed by Dunnett’s multiple comparison test, with WT as the reference sample (*p < 0.05 and **p < 0.01; N.S.: not significant). (B) ChIP-qPCR analysis of H3K9me2 and total H3 enrichment in WT, cid14Δ, pab2RRM3A, cid14Δpab2RRM3A, and pab2Δ. Data are presented as mean ± SD from three independent experiments. Statistical significance was determined using a one-way ANOVA followed by Dunnett’s multiple comparison test, with WT as the reference sample (*p < 0.05, **p < 0.01, ***p < 0.001, and ****p < 0.0001; N.S.: not significant). (TIF) [file pgen.1011647.s003.tif]

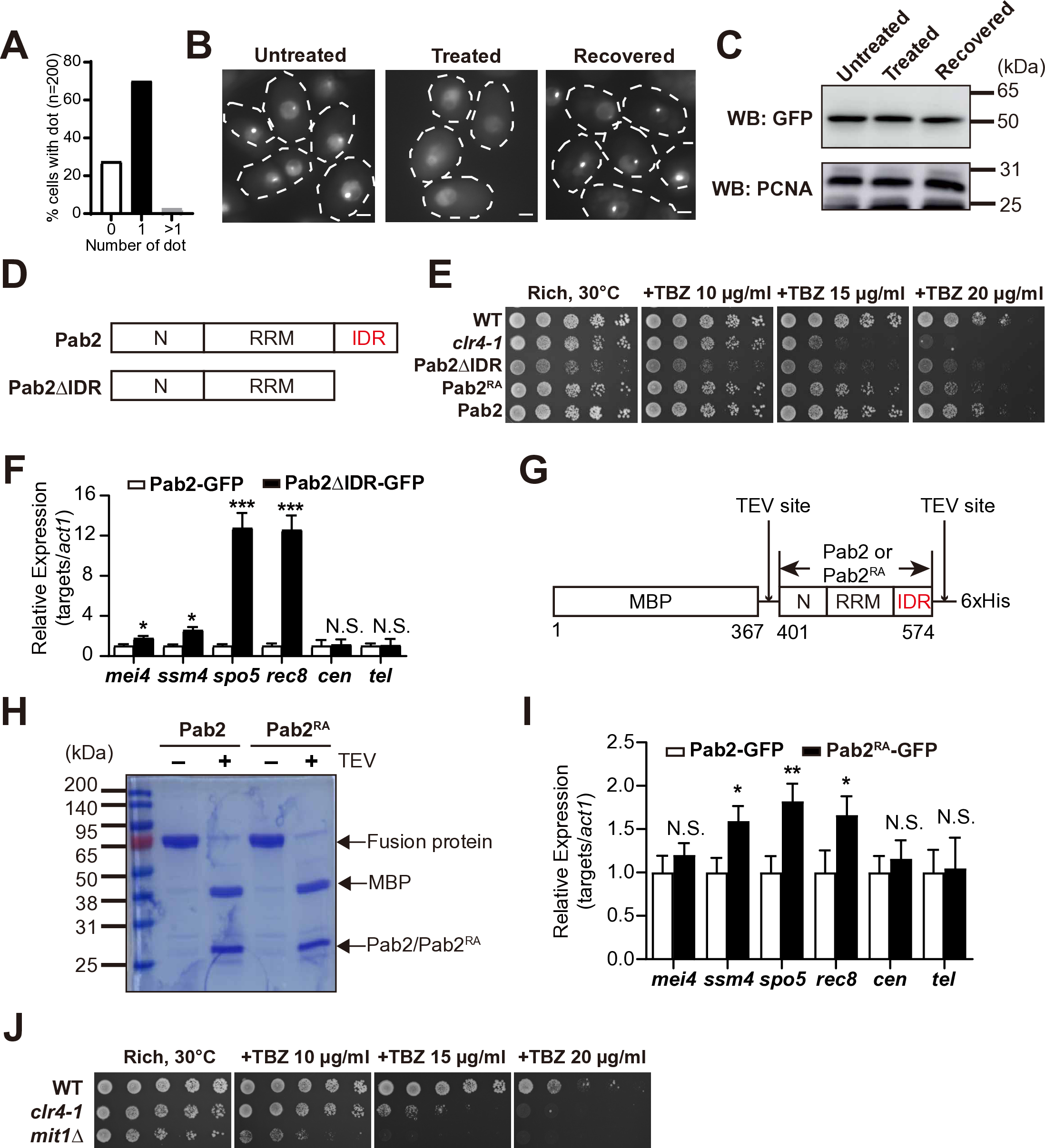

Supplement: S4 Fig — (A) The number of Pab2 foci in vegetative cells expressing Pab2-GFP. A total of 200 cells were examined. (B) Pab2-GFP localization in untreated, treated with 5% (w/v) 1,6-hexanediol for 10 minutes (Treated) and after a 60-min recovery period following 1,6-hexanediol removal (Recovered). Scale bars, 2 μm. (C) Western blotting of Pab2-GFP before, during, and after 1,6-hexanediol treatment (untreated, treated, and recovered). Pcn1/PCNA was used as a loading control. (D) Schematic representation of Pab2 and the Pab2ΔIDR truncation mutant proteins. (E) Thiabendazole (TBZ) sensitivity assay of untagged (WT), clr4–1, Pab2ΔIDR-GFP, Pab2RA-GFP, and Pab2-GFP cells. Ten-fold serial dilutions were spotted onto complete medium plates with or without TBZ and grown at 30°C. (F) RT-qPCR analysis of transcripts from mei4+, ssm4+, spo5+, rec8+, as well as centromeric (cen) and telomeric (tel) transcripts, in Pab2-GFP and Pab2ΔIDR-GFP strains. The transcripts were normalized to act1 mRNA to determine their relative expression levels. Data are presented as mean ± SD from three independent experiments. Statistical significance was determined using a two-tailed unpaired t-test (*p < 0.05 and ***p < 0.005; N.S.: not significant). (G) Schematic representation of MBP-Pab2–6 × His tag and MBP-Pab2RA-6 × His tag fusion proteins. (H) Purified MBP-Pab2–6 × His tag and MBP-Pab2RA-6 × His tag fusion proteins with and without TEV treatment were stained by Coomassie brilliant blue. (I) RT-qPCR analysis of transcripts from mei4+, ssm4+, spo5+, rec8+, as well as centromeric (cen) and telomeric (tel) transcripts, in Pab2-GFP and Pab2RA-GFP strains. The transcripts were normalized to act1 mRNA to determine their relative expression levels. Data are presented as mean ± SD from three independent experiments. Statistical significance was determined using a two-tailed unpaired t-test (*p < 0.05 and **p < 0.01; N.S.: not significant). (J) TBZ sensitivity assay of WT, clr4–1, and mit1∆ cells. Ten-fold serial [file pgen.1011647.s004.tif]

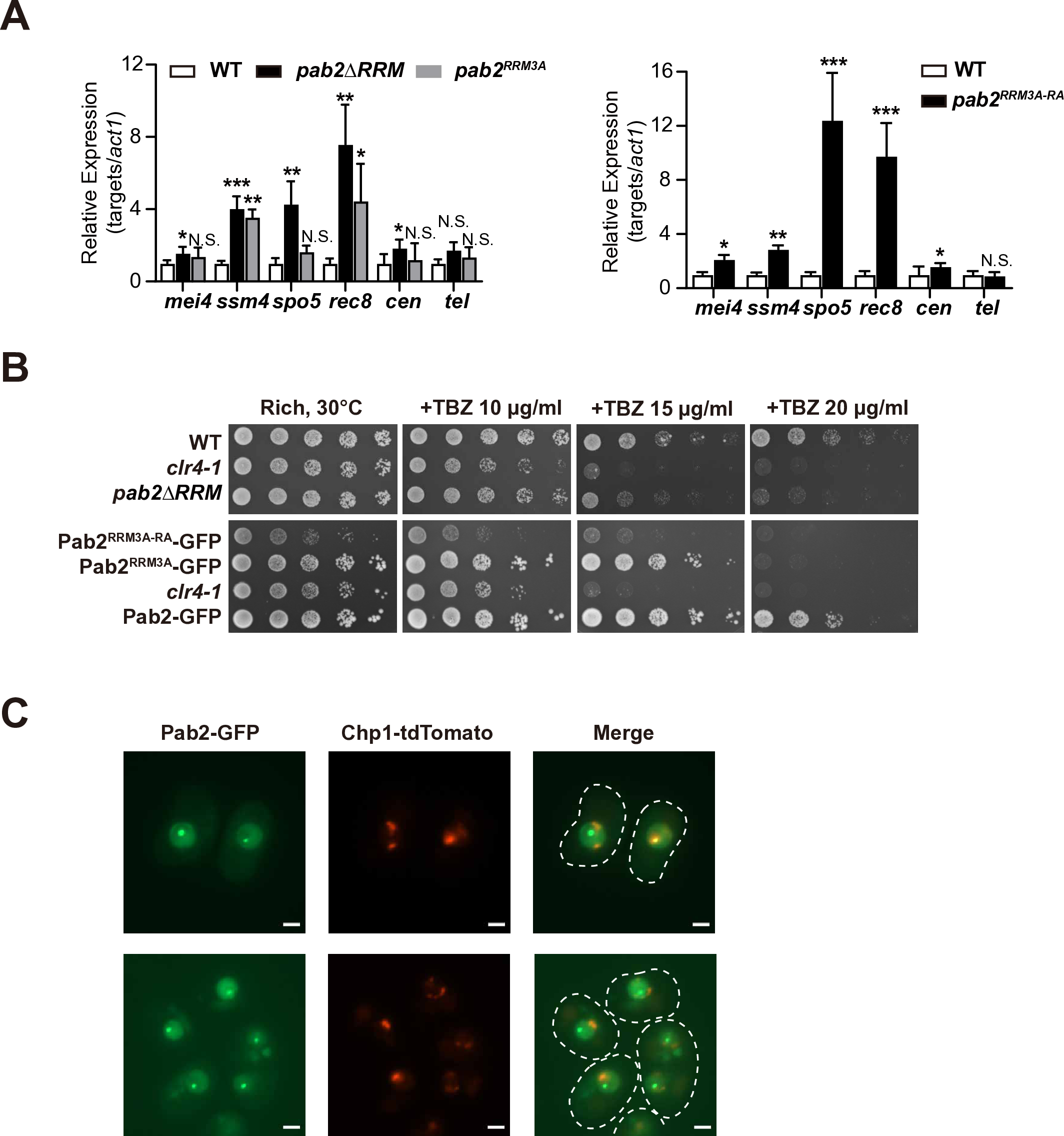

Supplement: S5 Fig — (A) RT-qPCR analysis of transcripts from mei4+, ssm4+, spo5+, rec8+, as well as centromeric (cen) and telomeric (tel) transcripts, in WT, pab2ΔRRM, pab2RRM3A, and pab2RRM3A-RA strains. The transcripts were normalized to act1 mRNA to determine their relative expression levels. Data are presented as mean ± SD from three independent experiments. (Left) Statistical significance was determined using a one-way ANOVA followed by Dunnett’s multiple comparison test, with WT as the reference sample (*p < 0.05, **p < 0.01, and ***p < 0.001; N.S.: not significant) (Right) Statistical significance was determined using a two-tailed unpaired t-test (*p < 0.05, **p < 0.01 and ***p < 0.005; N.S.: not significant). (B) Thiabendazole (TBZ) sensitivity assay of untagged (WT), clr4–1, pab2ΔRRM, Pab2RRM3A-RA-GFP, Pab2RRM3A-GFP, and Pab2-GFP cells. Ten-fold serial dilutions were spotted onto complete medium plates with or without TBZ and grown at 30°C. (C) Fluorescent microscopy of Pab2-GFP and Chp1-tdTomato localization during vegetative growth. Scale bars, 2 μm. (TIF) [file pgen.1011647.s005.tif]

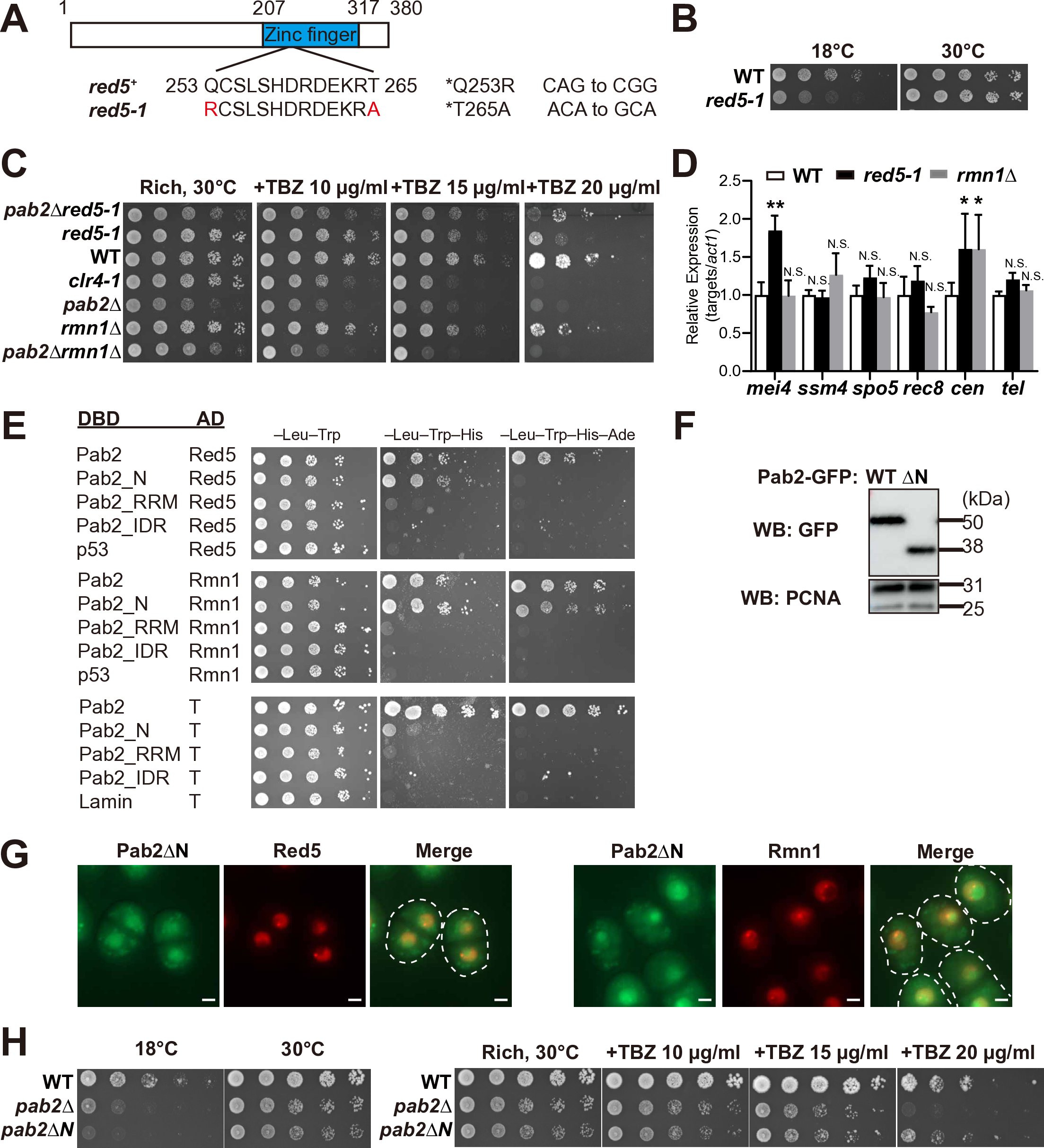

Supplement: S6 Fig — (A) Schematic representation of the Red5 protein and the mutations found in red5–1. Red5 has multiple CCCH-type zinc-finger motifs. The red5–1 allele contains two amino acid substitutions, Q253R and T265A, in the zinc finger domain. (B) Growth assay of wild-type (WT) and red5–1 cells. Ten-fold serial dilutions were spotted onto complete medium plates and incubated at 18°C and 30°C. (C) Thiabendazole (TBZ) sensitivity assay of the indicated strains. Ten-fold serial dilutions were spotted onto complete medium plates with or without TBZ and grown at 30°C. (D) RT-qPCR analysis of transcripts from mei4+, ssm4+, spo5+, rec8+, as well as centromeric (cen) and telomeric (tel) transcripts, in WT, red5–1, and rmn1Δ strains. The transcripts were normalized to act1 mRNA to determine their relative expression levels. Data are presented as mean ± SD from three independent experiments. Statistical significance was determined using a one-way ANOVA followed by Dunnett’s multiple comparison test, with WT as the reference sample (*p < 0.05 and **p < 0.01; N.S.: not significant). (E) The Pab2 protein was separated into three domains, the N-terminal domain (N), the RNA recognition motif (RRM), and the C-terminal intrinsically disordered domain (IDR), and each fragment was subjected to yeast two-hybrid assays to test its interaction with Red5 and Rmn1. (F) Western blotting of Pab2-GFP and Pab2ΔN-GFP. Pcn1/PCNA was used as a loading control. (G) The localization of Pab2ΔN-GFP/Red5-tdTomato and Pab2ΔN-GFP/Rmn1-tdTomato was examined by fluorescent microscopy. Scale bars, 2 μm. (H) Growth and TBZ sensitivity assay of WT, pab2∆, and pab2∆N strains. Ten-fold serial dilutions were spotted onto complete medium plates with or without TBZ and incubated at 18°C and 30°C (for growth assay) or at 30°C (for TBZ sensitivity assay). (TIF) [file pgen.1011647.s006.tif]

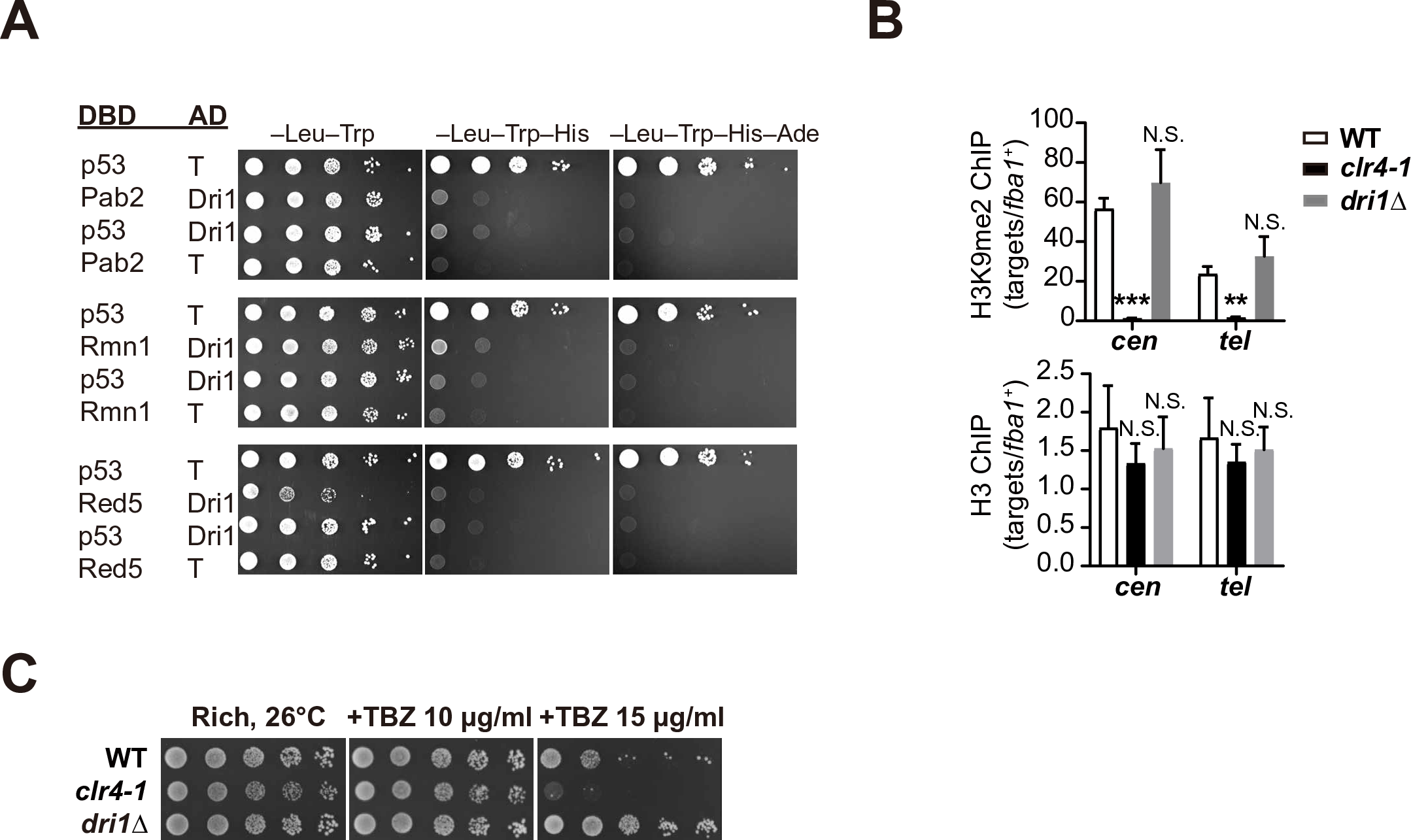

Supplement: S7 Fig — (A) Yeast two-hybrid assays to examine the interaction between Dri1 and Pab2. Ten-fold serial dilutions of the S. cerevisiae AH109 strain carrying indicated plasmids were spotted onto minimal plates lacking leucine and tryptophan (-Leu-Trp), leucine, tryptophan, and histidine (–Leu–Trp–His), or leucine, tryptophan, histidine, and adenine (–Leu–Trp–His–Ade). p53 and T antigen (T) were used as positive controls. (B) ChIP-qPCR analysis of H3K9me2 and total H3 enrichment at centromeres (cen) and telomeres (tel) using the indicated strains. Data are presented as mean ± SD from three independent experiments. Statistical significance was determined using a one-way ANOVA followed by Dunnett’s multiple comparison test, with WT as the reference sample (**p < 0.01 and ***p < 0.001; N.S.: not significant). (C) Thiabendazole (TBZ) sensitivity assay of the indicated strains. Ten-fold serial dilutions were spotted onto complete medium plates with or without TBZ and incubated at 26°C. (TIF) [file pgen.1011647.s007.tif]

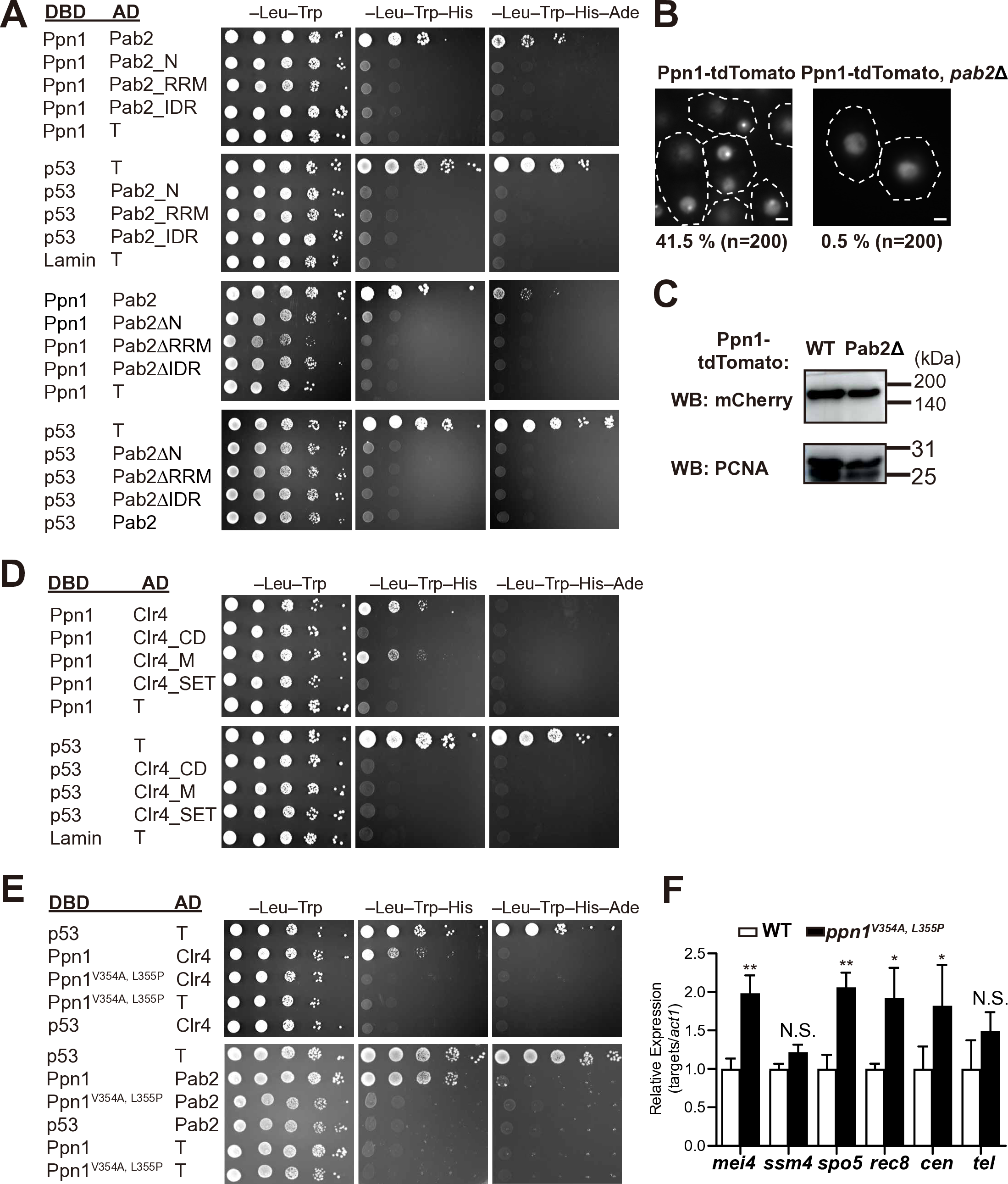

Supplement: S8 Fig — (A) Yeast two-hybrid assays testing the interaction between Ppn1 and the N-terminal (N), RNA recognition motif (RRM), intrinsically disordered domain (IDR), ΔN, ΔRRM, or ΔIDR of Pab2. Ten-fold serial dilutions of the S. cerevisiae AH109 strain carrying indicated plasmids were spotted onto minimal plates lacking leucine and tryptophan (–Leu–Trp), leucine, tryptophan, and histidine (–Leu–Trp–His), or leucine, tryptophan, histidine, and adenine (–Leu–Trp–His–Ade). p53 and T antigen (T) were used as positive controls. (B) The localization of Ppn1-tdTomato in vegetative WT and pab2Δ cells was examined by fluorescent microscopy. Scale bars, 2 μm. The percentage of cells which can form Ppn1 foci and the number of cells counted are shown below each panel. (C) Western blotting of Ppn1-tdTomato in WT and Pab2Δ strains. Pcn1/PCNA was used as a loading control. (D) Yeast two-hybrid assays for the assessment of the interaction between Ppn1 and three Clr4 fragments (CD: chromodomain, M: middle, and SET: the SET domain). The assays were performed as in (A). (E) Yeast two-hybrid assays for testing Ppn1-Clr4 and Ppn1V354A, L355P-Clr4 interactions. The assays were conducted as in (A). (F) RT-qPCR analysis of transcripts from mei4+, ssm4+, spo5+, rec8+, as well as centromeric (cen) and telomeric (tel) transcripts, in WT and ppn1V354A, L355P strains. The transcripts were normalized to act1 mRNA to determine their relative expression levels. Data are presented as mean ± SD from three independent experiments. Statistical significance was determined using a two-tailed unpaired t-test (*p < 0.05 and **p < 0.01; N.S.: not significant). (TIF) [file pgen.1011647.s008.tif]
