# Supplementary material for: The nuclear poly(A)-binding protein Pab2/PABPN1 promotes heterochromatin assembly through the formation of Pab2 nuclear condensates
Source: PLoS Genet. 2025 Mar 31;21(3):e1011647. doi: 10.1371/journal.pgen.1011647 (PMC12002642; doi:10.1371/journal.pgen.1011647)
Supplement: Attachment — Submitted filename: 250122_rebuttal.docx [file pgen.1011647.s023.docx]

**Reviewer #1:**

*1. About the spatial distribution of Pab2-GFP foci/condensates. The working model (Fig.7) seems to argue that these foci enrich the active form the Pab2 and CPF complexes, and would represent the site(s) for the actions. Extending this argument, it seems plausible that Clr4 protein and the centromeres (or other chromatin target loci) are brought to the site for heterochromatin assembly. Characterizing the possible co-localization (temporarily or constitutively) of centromeres and/or Clr4 with Pab2 foci would be a useful test of the model. Curiously, such characterization is missing.*

We thank Reviewer #1 for raising this important question regarding the spatial relationship between Pab2 and heterochromatin. This prompted us to conduct further experiments, leading to a refined understanding of Pab2's role. Our initial model (Fig 7 in the original manuscript) served as a starting point, but new data has allowed us to develop a more nuanced perspective. Specifically, co-localization analysis with Chp1-tdTomato, a marker for heterochromatin domains (S5C Fig), revealed minimal overlap with Pab2-GFP foci. This indicates that the large, microscopically visible Pab2 foci are not the primary sites of direct interaction with established heterochromatin.

However, this does not diminish Pab2's crucial role in heterochromatin assembly. Our ChIP-qPCR data consistently shows Pab2 association with centromeres (Figs 5F and 6H) and its requirement for proper Clr4 and Mit1 enrichment at these loci (Fig 4J). Therefore, we now propose that Pab2, potentially through smaller, less visible condensates or transient interactions, promotes the recruitment and/or retention of Clr4 and Mit1 at centromeres, facilitating heterochromatin assembly. The large Pab2 foci we observe likely represent sites of other Pab2 functions, such as meiotic mRNA decay, as evidenced by co-localization with Red1 (S1G and S1H Figs). This refined model is illustrated in the revised Fig 9, and the Discussion elaborates on the hypothesis that Pab2 forms two distinct types of condensates: one associated with meiotic mRNA decay (as indicated by Red1 co-localization) and another potentially involved in heterochromatin assembly.

*2. Also missing, is the genome-wide characterization of heterochromatin in pab2 mutants. A ChIP-seq analysis in addition to ChIP/qPCR that was used in the study would help probing whether other constitutive and/or facultative heterochromatin regions (the mating type locus? Meiosis gene loci?) are affected by pab2.*

We appreciate the reviewer's suggestion to characterize genome-wide heterochromatin in pab2∆ mutants. We have considered this carefully and addressed it through multiple lines of evidence.

First, published data from the Robin Allshire lab (GEO accession: GSM3070361, deposited by Tong P.), though not yet formally published, clearly demonstrates that H3K9me2 in S. japonicus is primarily localized to centromeres and telomeres. Unlike S. pombe, no evidence of distinct heterochromatin islands (e.g., at meiotic genes) was observed (see below screenshots). This existing data strongly suggests a fundamental difference in heterochromatin organization between these two species.

To further investigate the potential impact of pab2∆ on H3K9me2 distribution, we performed CUT&Tag analysis in wild-type and pab2∆ strains. Our results confirmed the centromeric (including the mat locus on centromere 3) and telomeric localization of H3K9me2, and the H3K9me2 was reduced in *pab2*∆. Consistent with the data from the Allshire lab, we did not detect significant H3K9me2 enrichment at syntenic loci in S. japonicus, either (please see below). To address the possibility that our CUT&Tag assay lacked the sensitivity to detect small heterochromatin islands, we performed targeted ChIP-qPCR at *mei4*^+^ and *ssm4*^+^, two meiotic gene loci. These experiments also failed to reveal significant H3K9me2 enrichment (Fig 3B).

Our CUT&Tag, as well as ChIP-seq by the Allshire lab (Tong P. 2019), detected H3K9me2 enrichment at tRNA/rRNA clusters on chromosome II. However, these signals are likely an artifact due to the high sequence similarity among tRNA and rRNA genes, which are also found within constitutive heterochromatin. Therefore, we believe that the observed H3K9me2 at these clusters is not indicative of bona fide heterochromatin domains but rather a technical artifact.

Taken together, these data, including published data from the Allshire lab, our CUT&Tag, and our targeted ChIP-qPCR, strongly suggest that, under our experimental conditions, S. japonicus lacks the distinct heterochromatin islands characteristic of S. pombe. We also like to point out that the biological significance of *S. pombe* heterochromatin islands, in particular, those at meiotic genes, is still controversial. While we cannot definitively rule out the existence of heterochromatin islands below the detection limit of our assays, the collective evidence makes this unlikely.

We acknowledge the potential interest in a comprehensive mapping of heterochromatin in S. japonicus. However, the primary focus of this study is to elucidate how Pab2 facilitates/maintains heterochromatin assembly. A comprehensive heterochromatin mapping project is beyond the scope of this manuscript. We believe the data presented here sufficiently addresses the reviewer's concern by demonstrating that pab2∆ does not detectably alter the major H3K9me2 domains in S. japonicus

*3. RNA/IP analysis has shown that the transcripts derived from cen and tel regions are associated with Pab2. If such analysis can be scaled up for RNA-seq, this would also help addressing the specificity or preference for target RNAs binding by Pab2.*

According to this suggestion, we have indeed performed RNA-IP followed by RNA-seq (RNA-IP-seq) once to further investigate Pab2's RNA binding specificity. Combining the RNA-IP-seq data using Pab2-GFP/Pab2 mutant-GFP and RNA-seq data using wild-type and *pab2*∆, we found that: (1) Pab2 binds to several RNA classes, including both centromeric RNA and meiotic mRNAs, whose expression levels are altered in the *pab2*∆, (2) Pab2 also binds to RNAs whose expression levels remain unchanged in *pab2*∆, and (3) there are RNAs whose expression is affected by *pab2*∆ but do not show detectable Pab2 binding. Based on these findings, however, we are afraid that RNA-IP-seq would not significantly enhance our understanding of either Pab2's role in heterochromatin assembly, which is the central focus of this study, or Pab2 specificity. Therefore, we decided not to include the RNA-IP-seq data, but we included additional RNA-IP-RT-qPCR results (Fig 2D). These results indicate that Pab2 preferentially associates with centromeric RNA and three meiotic mRNAs, but it does not with two mRNAs derived from *tbp1*^+^ and *cdc2*^+^. Given its general affinity for poly(A) tails, it is unlikely that Pab2 associates with every mRNA in the cell, suggesting that Pab2 exhibits a degree of target specificity. These results also suggest that Pab2-binding to a transcript could be a prerequisite but not sufficient for heterochromatin formation. The mechanisms underlying this specificity are currently unknown, but it is plausible that Pab2-binding proteins, such as the Zn-finger protein Red5 and the RRM-containing protein Rmn1, contribute to RNA target selection, as suggested by our new results shown in Fig 6. While we acknowledge the value of identifying the specificity-determinant of heterochromatin assembly, we believe it falls outside the scope of the present work, which focuses specifically on Pab2's role in heterochromatin assembly.

*4. Fig.2C and other relevant figures. It is not totally clear whether H3K9me2 ChIP/qPCR in clr4-1 was done at permissive or restrictive temperature. This should be clarified, which would allow deducing that severe reduction in cen-heterochromatin compromises cell division or not under non-challenged condition.*

We apologize for omitting this detail. The H3K9me2 ChIP/qPCR experiments in clr4-1, as shown in Fig 2C and other relevant figures, were conducted at the permissive temperature of 26°C. We chose this condition to minimize potential secondary effects arising from cell death at the restrictive temperature. This information has now been included in the Materials and Methods.

*5. Page 18 Line 297 “we randomly mutated the pla1+ gene and identified the pla1-3 …” – this is unclear in the sense that how the pla1-3 mutant was identified? Based on a specific phenotype such as ts or hypersentivity to TBZ? Explanation about how to isolate. Likewise, Page 19 Line 330 “we first constructed a red5-1 mutant” – authors should explain how the point mutations were chosen or identified.*

We apologize for the lack of detail. The pla1-3 and red5-1 mutants were identified through a screen for temperature-sensitive (ts) mutants exhibiting defects in heterochromatin assembly. Specifically, ts candidates were screened for sensitivity to TBZ (thiabendazole) and reduced H3K9me2 levels by ChIP-qPCR. The mutants exhibiting the most pronounced phenotypes (TBZ hypersensitivity and reduced H3K9me2) were selected for further analysis. Details on the mutant isolation procedure have been added to the Materials and Methods section.

*6. Line 406-407. This section is hard to follow. I am not clear how the genetic suppression of ppn1D lethality by leo1D is directly relevant to the main story line here. Perhaps consider removing this section or moving it to Supplementary Info.?*

We thank Reviewer #1 for pointing out the lack of clarity regarding the interaction between ppn1∆ and leo1∆. We understand the concern that this might seem unrelated to the main focus. However, this experiment is essential to justify how we study Ppn1 in S. japonicus. A key difference between S. japonicus and S. pombe is that S. japonicus requires RNAi to survive (PMID: 35226668), while S. pombe does not have this requirement (PMID: 12193640). This means that in S. japonicus, trying to remove one of these essential RNAi components, such as Dcr1/Dicer, is lethal to the cell. When we tried to delete ppn1 in wild-type S. japonicus cells, we were unable to do so. This suggested two possibilities: either we had a technical problem with the deletion process, or ppn1^+^ is indeed essential for survival in S. japonicus. To distinguish between these possibilities, we tried deleting ppn1 in cells where another gene, leo1, had already been deleted. We successfully obtained cells lacking both ppn1^+^ and leo1^+^. This result demonstrates that the lethality caused by removing ppn1^+^ is dependent on leo1^+^. Since we couldn't simply remove ppn1 on its own, we decided to generate a temperature-sensitive version of ppn1 (ppn1ts). Thus, it would be difficult to explain why we used a ppn1ts mutant instead of ppn1∆ without explaining the *leo1*∆ suppression result.

Still, we acknowledge that the section may not look related to the main focus, as Reviewer#1 pointed out. To avoid interrupting the flow of the main narrative with a lengthy explanation of ppn1 essentiality, we have condensed this section as shown below and combined it with the subsequent section describing the effects of Ppn1 mutations on H3K9me2 levels.

“To investigate Ppn1's role in heterochromatin formation in S. japonicus, we initially attempted to generate a *ppn1*∆ strain. Although ppn1^+^ is non-essential for viability in S. pombe [75], we were unable to obtain a viable *ppn1*∆ strain in a wild-type S. japonicus strain. This observation, while requiring further investigation, hints at a potential difference in Ppn1 function or genetic context between the two species, possibly involving RNAi-dependent heterochromatin assembly as suggested by previous reports on RNAi components in S. japonicus [23].”

*7. Line 604 “S.japonicus may be better than S.pombe as a model organism for studying cell biology an chromosome biology” – this statement seems overly provocative. Authors might want to consider toning it down such as “S.japonicus has its unique strength in/be more suited for studying ……”*

We agree with Reviewer #1 that our original claim may have been overstated. We have therefore revised the text to to moderate our claim as suggested. For example, we wrote, “Furthermore, consistent with previous reports [23, 39, 72], our findings underscore that *S. japonicus* possesses unique characteristics that make it suitable for studying chromosome biology.”

*8. In Discussion, some relevant information was described as “data not shown”. It seems that some, in particular, Ser2 phosphorylation of Pab2 and Pab2 mutations (F101A, Y103A; G82T, I84R) could be presented in main text together with other Pab2 domain characterizations.*

We thank Reviewer #1 for this suggestion. We had initially investigated the effects of mutations in the Pab2 RRM domain (Pab2^F101A/Y103A^ and Pab2^G82T/I84R^) on heterochromatin assembly. However, the Pab2^F101A/Y103A^ mutant, despite mutations in the RNP1 consensus sequence, retained the ability to bind centromeric RNAs and showed no significant reduction in H3K9me2 levels (our unpublished observation). Because these results were inconclusive regarding the role of RNA binding in heterochromatin assembly, we opted not to include them in the main text.

To further probe the role of RNA binding, we generated a triple mutant, Pab2^Y61A/F101A/Y103A^, carrying the three substitution in both the RNP1 and RNP2 consensus sequences. This triple mutant exhibited phenotypes similar to the pab2∆ strain, including sensitivity to cold and TBZ and reduced H3K9me2 levels, and loss of RNA binding (Figs 5D and 5E and S5B Fig). These results provide compelling evidence for the importance of Pab2's RNA binding activity in heterochromatin assembly, strengthening our conclusions. Therefore, we have removed the description of the inconclusive RNP1 single/double mutants from the manuscript and now present the more informative triple mutant data in Fig 5 and S5 Fig.

Regarding the Pab2 Ser2 mutants (Pab2^S2A^ and Pab2^S2E^) and Pab2^G82T/I84R^, these analyses are ongoing and will be presented in a separate publication, as they are not essential for the conclusions of the current study.

**Reviewer #2:**

*1. In “Author Summary”, the authors do not mention that this work is done using S. Japonicus, just mentioning “fission yeast”. It is confusing for the readers.*

As suggested by Reviewer #2, we have clarified in the Author Summary that this work was performed using S. japonicus as below:

“In this study, we investigate the role of the nuclear poly(A)-binding protein Pab2 (human PABPN1 ortholog) in heterochromatin formation in the fission yeast *Schizosaccharomyces japonicus*.”

*2. In Introduction, authors do not mention about the previous research that tried to address the role of RNAi in constitutive heterochromatin formation in S. japonicus (ref 31). I feel relationship between RNAi and Pab2 is an important issue in this manuscript. So, authors should mention about it in introduction for easy understanding.*

We appreciate Reviewer #2 bringing this point to our attention. We have taken this opportunity to substantially revise the Introduction part. It now provides a more thorough overview of heterochromatin, including: key findings from *S. pombe* studies, current knowledge of heterochromatin in *S. japonicus*, and the potential connection between polyadenylation and heterochromatin/RNAi. This revised Introduction offers a clearer context for our study and highlights the importance of the relationship between RNAi and Pab2.

*3. Line 147, Fig. 2A: authors claimed that RNA levels from matin-type locus genes (SJAG_05153, SJAG_05154, and SJAG_01113) was increased but I could not see the increase in Fig. 2A.*

We thank the reviewer for pointing out this discrepancy. We have clarified the text to accurately reflect the data. While we previously stated that all mating-type locus transcripts were increased, our data (now clarified) shows that transcript levels of mat locus genes (SJAG_05153, SJAG_05154, and SJAG_01113) are increased in pab2∆ cells, but not in clr4-1 cells. This observation is consistent with a previous study in S. pombe showing increased mei4 mRNA in pab2∆ but not clr4∆ cells (PMID: 22144463), suggesting a conserved role for Pab2 in the degradation of mRNA derived from genes embedded within heterochromatin.

Reviewer#2 correctly notes that the greater reduction of H3K9me2 in clr4-1 might lead one to expect increased transcripts from the *mat* locus. Our findings, however, support a model where RNA degradation, rather than heterochromatin-mediated transcriptional silencing, is the primary mechanism controlling mat locus transcript levels. We have now included RT-qPCR analysis (Fig. 2C) demonstrating that mat locus transcripts accumulate in pab2∆ and red1∆ cells, but not in clr4-1. This supports our hypothesis that Red1, which is dispensable for heterochromatin assembly in S. japonicus, promotes mat transcript degradation, and that this RNA degradation pathway (dependent on Pab2 and Red1) remains functional in clr4-1 cells, despite the reduced H3K9me2.

*4. Figure legend of Fig. 2A: authors mentioned the figure represents centromeric and telomeric regions, but it included the data of mating-type locus genes.*

We thank the reviewer for this important clarification. We apologize for the oversight in the original figure and legend. We recognize that the location of the mating-type (mat) locus within the pericentromeric heterochromatin of chromosome III in S. japonicus is a key difference from its distal location in S. pombe. To address this, we have made the following changes to Figure 2: (1) we have added "Centromere 3R" to indicate the right arm of chromosome 3 on the schematic; and (2) we have revised the figure legend to explicitly state "the mating-type locus (located within the pericentromeric heterochromatin of chromosome III)." These changes will ensure clarity and avoid future confusion.

*5. In Figures of H3K9me2-ChIP-PCR experiments, authors described the position of the primes just as “cen” and “tel”. Centromeres and telomers are large region and authors should describe or show the positions of the primers more clearly.*

We thank the reviewer for raising this important concern. We apologize for not specifying the primer locations more clearly in the original manuscript. To address this, we have included a new figure (please see S2A Fig) that depicts the genomic context of centromere 3 and telomere 2R, along with the precise locations of the cen and tel amplicons used in our qPCR assays.

*6. Figures 4F: y-axis of the graph represented the ratio between control RNA and cen RNA. What is the control RNA and cen RNA. Did cen RNA represent RNA from centromeric RNA?*

We thank the reviewer for this important clarification. We apologize for the lack of clarity in the original figure legend. In Fig 4F (now shown as Figure 5D), the y-axis represents the relative enrichment of centromeric RNA (cen) normalized to act1 mRNA. This same normalization method was used for all other relevant RNA-IP figures, and we have revised those figure legends for clarity and consistency.

*7. Lines 484-494, lines 495-504: At this part, authors discuss a few important points using unpublished data. For readers, it is hard to evaluate their discussion without the real data. Authors should shorten their arguments about these points.*

We thank Reviewer #2 for this valuable comment. We agree that discussing unpublished data weakened our argument. Therefore, we have significantly revised the discussion to remove all the unpublished data regarding the various Pab2 RRM domain mutants (Pab2^F101A/Y103A^, Pab2^G82T/I84R^, Pab2^S2A^, and Pab2^S2E^). To address this point further, we performed additional experiments using a Pab2 RRM triple mutant strain (Pab2^Y61A/F101A/Y103A^) we generated recently. The revised discussion now focuses solely on the data obtained from the triple mutant, presented in Fig 5 and S5 Fig, which demonstrates the crucial role of Pab2's RNA binding activity in heterochromatin assembly.

**Reviewer #3:**

Major points:

*1- There is no evidence that Pab2 nuclear dots effectively colocalize with constitutive heterochromatin. This is crucial point as the whole model is based on this assumption. In fact, it appears that most cells contain a unique Pab2 dot, which is not what might be expected for heterochromatin localization. An alternative possibility could be that Pab2 coalesces in dots, together with factors involved in meiotic mRNA decay (i.e. MTREC components), which could rather overlap with euchromatic loci, as in S. pombe.*

We thank Reviewer #3 for raising the important question of Pab2-GFP localization in relation to heterochromatin. We agree that understanding the spatial relationship between Pab2 and heterochromatin components is crucial for our model. We have carefully considered this point and have performed experiments that shed light on this complex relationship. We first investigated whether Pab2 dots are associated with heterochromatin or components of the meiotic mRNA decay machinery MTREC. Our microscopic analyses demonstrated that Pab2 hardly colocalizes with Chp1, a chromodomain protein in the RITS complex that binds to H3K9me2 but colocalizes with the MTREC component Red1 (S1H and S5C Figs), indicating that Pab2 dots are related to meiotic mRNA decay. We next assessed whether Pab2 binds to heterochromatin. To this end, we performed Pab2-GFP chromatin-IP and found that Pab2 is indeed associated with pericentromeric heterochromatin, and this association is dependent on Pab2 RRM and IDR (Fig 5F). Based on these combined observations, we propose that Pab2 is involved in both meiotic mRNA decay, as suggested by its colocalization with Red1, and is also present at centromeres, suggesting a role in centromere function and/or heterochromatin organization. Thus, our work provides a more comprehensive understanding of Pab2's multifaceted roles. However, we acknowledge that our original model placed greater emphasis on a direct role of Pab2 dots in heterochromatin formation, and we revised the model to reflect our new results (Fig 9).

*The authors should also provide the percentages of cells containing zero, one or more nuclear foci.*

We thank Reviewer #3 for this suggestion. We have analyzed the number of Pab2 dots per cell and have now included the percentages of cells containing zero, one, or more than one focus in S4A Fig. Specifically, we found that 27.5% of cells contained zero foci, 70% contained one focus, and 2.5% contained more than one focus.

*2- Pab2 is proposed to associate with centromeric transcripts and CPF to in turn recruit Clr4. This is not supported by data. If correct, the recruitment of Clr4 and/or heterochromatin factors requiring H3K9me2 for localization should be decreased in Pab2, MTREC (i.e. Red5, Rmn1) and CPF (e.g. Ppn1) mutants. Pab2, MTREC and CPF should also themselves be present at heterochromatic loci, which can be assessed by ChIP.*

We thank Reviewer #3 for this comprehensive suggestion. We understand that all the ChIP experiments you suggested would provide further insights, but we believe the most critical experiment that supports our proposed model is Clr4 ChIP in wild-type and pab2∆. We generated a strain expressing N-terminally 3×FLAG-tagged Clr4 (5×FLAG-Clr4) by tagging the endogenous clr4^+^ gene at its N-terminus and performed Clr4 ChIP. We found that Clr4 did associate with centromeres in wild-type, but the association was significantly reduced in pab2∆ (Fig 4J), supporting our idea that Pab2 facilitates Clr4 recruitment to centromeres. We also attempted ChIP-qPCR of Red5, Rmn1, and Ppn1, all of which are involved in H3K9 methylation, but we did not observe any significant enrichment of these proteins at centromeres even in the presence of the long crosslinker dimethyl adipimidate dihydrochloride (DMA), suggesting that these proteins do not form stable or readily detectable interactions with chromatin at these loci using our ChIP assay. Instead, we found that Mit1, a chromatin remodeler essential for heterochromatic silencing, is associated with centromeres in a Pab2-dependent manner (Fig 4J). Also, S. japonicus Mit1, unlike S. pombe Mit1, is critical for H3K9 methylation (Fig 4K). While we could not perform all the ChIP experiments suggested by the reviewer, our findings on Clr4 and Mit1 localization provide compelling evidence for the importance of Pab2 in heterochromatin assembly and address the core concern of Pab2's role in Clr4/Mit1 recruitment, as illustrated in our model (Fig 9).

*The RNA-IP of Pab2 to centromeric RNAs (Fig 2F) could reflect binding to a fraction of transcripts that have been released in the nucleoplasm.*

We thank Reviewer #3 for raising this important point regarding the interpretation of our RNA-IP data (Fig 2F in the original manusript). We acknowledge the possibility that the detected Pab2-centromeric RNA interaction could, in theory, reflect binding to a fraction of transcripts released into the nucleoplasm. However, several lines of evidence strongly suggest that the Pab2-bound centromeric RNAs detected in our RNA-IP experiments are primarily localized within the nucleus: (1) our microscopic analyses consistently show that Pab2 is predominantly localized within the nucleus (e.g., S1H Fig and Fig 4C). This predominantly nuclear localization makes it less likely that a significant portion of Pab2 is interacting with released centromeric RNAs in the nucleoplasm; (2) importantly, we observed that Pab2 mutants deficient in RNA binding (e.g., Pab2^Y61A/F101A/Y103^, also see our response to comment 5) are mislocalized to the cytoplasm or diffusive in the nucleus (Fig 5C). This observation strongly suggests that RNA binding is required for Pab2's nuclear localization. If Pab2 were primarily binding released transcripts in the nucleoplasm, we would not expect to see such a dramatic shift to cytoplasmic localization when RNA binding is disrupted; and (3) it is well established that the TRAMP complex and other RNA degradation factors involved in the processing and turnover of non-coding RNAs, including centromeric RNAs, are primarily localized and function within the nucleus (S3D Fig). This further supports the idea that the majority of centromeric RNA degradation occurs within the nuclear environment. Therefore, we believe that our RNA-IP data primarily reflects Pab2 binding to centromeric RNAs within the nucleus, rather than to released transcripts in the nucleoplasm.

*3- The absence of Pab2 results in a minor accumulation of centromeric and telomeric transcripts (Fig 2A, 2B), especially when compared to the clr4-1 mutant (the different scales used in upper panels of Fig 2A to compare wt, pab2∆ and clr4-1 strains are misleading in this respect). This might suggest that, despite being reduced, H3K9me2 levels in pab2∆ cells suffice to maintain most of the silencing activity and questions about the relative importance of Pab2 in promoting the assembly of functional heterochromatin domains. These aspects should be clearly mentioned in the manuscript.*

We thank Reviewer#3 for this important point. We acknowledge that the minor accumulation of centromeric and telomeric transcripts in pab2∆ cells (Fig 2A and 2B), especially when compared to the clr4-1 mutant, raises questions about the relative importance of Pab2 in heterochromatin assembly. We believe this subtle change is due to the presence of redundant post-transcriptional silencing mechanisms, as reported in *S. pombe*. Specifically, while Pab2 may contribute to silencing, other pathways, such as RNAi and the TRAMP complex, are still functional in pab2Δ cells, thus limiting the accumulation of transcripts. To test this hypothesis, we examined the role of the TRAMP complex, represented by the non-canonical poly(A) polymerase Cid14. RT-qPCR analysis of centromeric and telomeric transcripts in cid14Δ cells showed elevated transcript levels (S3A Fig), indicating that the TRAMP complex contributes to the post-transcriptional control of these transcripts. To examine whether Cid14 and Pab2 function in the same pathway, we attempted to generate a cid14Δpab2Δ double deletion strain. However, we were unable to obtain this double mutant (data not shown), suggesting that the combined loss of both pathways leads to a critical reduction in heterochromatin, resulting in synthetic lethality and preventing cell viability.

Instead, we successfully generated cid14Δpab2^RRM3A^ (please see our response to Major point 5) and found that the decrease in H3K9me2 at centromeres and telomeres in this double mutant was more severe than that of cid14Δ or pab2^RRM3A^ (S3B Fig). These results indicate that Cid14 (or TRAMP) works in a distinct pathway to suppress centromeric transcripts at the post-transcriptional level, thus explaining the minor change in cen/tel transcripts observed in pab2Δ. Consistent with this idea of post-transcriptional control, we observed a significant loss of H3K9me2 without a corresponding significant increase in transcript upregulation in several other pab2 mutants (e.g., Pan2∆N), further supporting the notion that changes in H3K9me2 levels do not directly translate into proportional changes in transcript abundance due to post-transcriptional silencing mechanisms.

We understand that using different scales in the panels in Fig 2A to compare WT, *pab2*Δ, and *clr4-1* makes direct visual comparison challenging. However, visualizing the subtle but statistically significant upregulation in *pab2*Δ is essential to demonstrate that Pab2 does indeed have a role in gene silencing, albeit a minor one relative to Clr4. Using a single scale would obscure this important finding. Therefore, we have prioritized visualizing this subtle change by using different scales. We have clearly indicated the scale for each panel in Fig 2A and stated it in the figure legend: “Note that the *clr4-1* panels use a different scale for normalized reads (RPM).”

*It is also unclear why the authors did not systematically analyzed the levels of centromeric transcripts in the various mutants of interest (e.g. pab2RRM∆;IDR∆;RA, rmn1∆, red5-1, ppn1V354A,L355P) and compared these to clr4-1.*

We thank Reviewer #3 for this important suggestion. While our initial analysis indicated that the increase in centromeric transcript levels in the mutants of interest was substantially less pronounced than that observed in clr4-1, suggesting a more subtle contribution of these factors to centromeric silencing, we have now included those RT-qPCR analyses. These results are presented in Figs 6G and 7D and S4F, S4I, S5A, S6D, and S8F Figs.

*4- Throughout the paper, evidence for heterochromatin defects in the various mutants tested is solely based on H3K9me2 ChIP. The authors should perform additional ChIP experiments to evaluate total H3 (as a control), H3K9me3 as well as RNAPII levels. This could give further insights about the steps at which the different factors act. ChIP H3 and H3K9me3*

We thank Reviewer #3 for this important suggestion. As requested, we have performed additional ChIP experiments. Histone H3 ChIP was performed to confirm consistent nucleosome occupancy across the tested mutants. H3K9me3 ChIP was conducted using the anti-H3K9me3 antibody (Affinity Biosciences, Cat#DF6938), which showed the most consistent and specific signal in our initial tests. We observed a significant reduction of H3K9me3 at centromeres in pab2Δ and clr4-1, but not at telomeres (Fig 3A). RNA Polymerase II (Pol II) ChIP results are now included in S2F Fig.

*5- Data about Pab2 RRM point mutants should be shown in the manuscript (not only mentioned in the discussion) to appreciate the relative contribution of RNA-binding per se. Since the associated phenotypes are described as weaker than RRM∆, this questions about the importance of RNA-binding. It could be that Pab2 is structurally disrupted in the absence of the RRM and hence cannot associate with Ppn1. This could be tested in vivo by coIP. The authors could also combine Pab2 RRM and IDR point mutans (e.g. pab2F101A,Y103A,RA) to test whether RNA-binding is abolished or not.*

We thank Reviewer #3 for this suggestion. We agree that understanding the relative contribution of RNA binding is crucial. Since our initial RRM mutant (Pab2^F101A/Y103A^) did not show obvious phenotypes, we understand that the description in the discussion could have led to confusion regarding the importance of RNA binding. Also, using the Pab2∆RRM strain raised questions about potential structural disruptions affecting Ppn1 association.

To address these concerns and to avoid potential artifacts associated with large-scale structural changes that might occur upon complete deletion of the Pab2 RRM*,* we generated a new RRM mutant strain (Pab2^Y61A/F101A/Y103^) as well as a combined mutant carrying amino acid substitutions in both the RRM and IDR (Pab2^RRM 3A-RA^). Unlike a full RRM deletion, these point mutations are less likely to cause gross structural changes in Pab2, allowing us to more specifically assess the role of the RRM in RNA binding and other functions.

Our experiments revealed the following: First, Pab2^Y61A/F101A/Y103A^ cannot form nuclear dots, thought it can localize to the nucleus (Fig 5C). Second, the ability of Pab2^Y61A/F101A/Y103A^ is significantly compromised compared to that of Pab2WT (Fig 5D). Third, Pab2^RRM 3A-RA^, which does not associate with centromeric RNAs, cannot localize to the nucleus (Fig 5C and 5D). Forth, the reduction in H3K9me2 was most evident in Pab2^RRM 3A-RA^ among various *pab2* mutants (Fig 5E). Finally, Pab2 binding to pericentromeric heterochromatin was decreased to the background level in the Pab2^RRM 3A-RA^ strain but not in the other *pab2* mutant strains (Fig 5F). These data, particularly the observation that Pab2^Y61A/F101A/Y103^ maintains nuclear localization but loses condensate formation and the combined mutant shows severely compromised RNA association, strongly suggest that the RRM is directly involved in RNA binding and condensate formation and not simply required for protein stability or interaction with Ppn1 due to gross structural changes. Therefore, given these results with the point mutants, a co-IP experiment using Pab2ΔRRM is less critical for addressing the role of the RRM in Pab2 function.

*6- The formation of Pab2 and Ppn1 nuclear dots is shown to depend on specific residues/domains (Fig 3C, 3F, 4C, S6F) but are these mutations affecting the localization of the protein partner (i.e. Pab2-GFP in ppn1V354A,L355P cells and Ppn1-tdTomato in pab2∆ cells)? This may provide some insights about the order of events.*

We thank Reviewer #3 for this important question, which prompted us to further clarify the relationship between Pab2 and Ppn1. To address whether the absence of Pab2 affects Ppn1 localization, we generated a pab2∆ strain expressing Ppn1-tdTomato. Western blotting confirmed that the steady-state level of Ppn1-tdTomato in pab2Δ was comparable to that in WT (S8B Fig). However, our microscopic analyses demonstrated that Ppn1 dot formation was almost completely abolished in pab2Δ cells (0.5% compared to 40.5% in WT), as shown in S8C Fig. This observation strongly suggests that Pab2 is required for Ppn1 dot formation, supporting the model that Pab2 recruits Ppn1 to the site where Pab2 works for heterochromatin assembly and mRNA degradation. Thus, our analyses strongly support a functional link between Pab2 and Ppn1.

We also attempted to construct a ppn1^V354A/L355P^ strain expressing Pab2-GFP using both a standard genetic crosses and transformation. However, we have been unable to obtain such a strain so far. This failure to obtain the double mutant suggests a synthetic lethal interaction between the ppn1^V354A/L355P^ mutation and the expression of Pab2-GFP. This synthetic lethality may indicate that the combination of these genetic perturbations leads to a critical defect, possibly in heterochromatin structure or function.

*Other specific points:*

*- meiotic mRNAs accumulate in pab2∆ cells (Fig 1). Are they enriched in distinct cis-motifs, similar to what seen in S. pombe (UNAAAC motifs recognized by Mmi1)?*

We thank Reviewer #3 for raising this interesting question. To investigate whether the accumulated meiotic mRNAs in pab2Δ cells (Fig 1) are enriched in DSR-like cis-motifs, we searched for the hexanucleotide sequences UUAAAC and UCAAAC (known Mmi1 binding motifs in S. pombe) in the S. japonicus mRNAs of mei4, mcp5, and the highly affected gene SJAG_01096. Our analysis revealed the following: S. japonicus mei4 mRNA contains one UUAAAC and one UCAAAC motif, while S. pombe mei4 mRNA has four UUAAAC and four UCAAAC motifs. Similarly, S. japonicus mcp5 RNA carries one UUAAAC and one UCAAAC hexanucleotide sequence, whereas S. pombe mcp5 mRNA contains seven UUAAAC and five UCAAAC sequences. The highly affected gene SJAG_01096 includes just one UCAAAC but no UUAAAC motif. Based on these findings, U(U/C)AAAC motifs can also be found in S. japonicus meiotic mRNAs. However, similar to observations in S. pombe, the number of these motifs does not correlate with the observed changes in expression levels when the RNA degradation pathway is compromised. This suggests that these motifs are not the primary drivers of mRNA accumulation in pab2∆ cells.

Alternatively, assuming the Mmi1-Pab2 functional link is conserved in S. japonicus, the differences in the YTH domain sequence (which is responsible for RNA binding and shows ~66% identity and ~85% similarity between S. japonicus and S. pombe Mmi1) could potentially alter the RNA binding specificity of Mmi1. These differences in the YTH domain sequence could potentially alter the RNA binding specificity of Mmi1. Further analyses, such as RNA IP-seq, are required to definitively address this point.

*- In Fig S1d, no representative image is shown for the mating/sporulation defects of pab2∆ cells.*

We thank Reviewer #3 for this suggestion. We have now included representative images of sporulation in WT and pab2Δ cells in S1D Fig. These images show a reduction in the number of asci formed in pab2Δ cells compared to WT, confirming the mating defect shown in S1E Fig.

*- In Fig 2A, clr4-1 mutant does not accumulate transcripts from the mating type locus. What is the rationale for this observation?*

We thank Reviewer #3 for this important question. A previous study demonstrated that the mei4+ locus is coated by H3K9me2 during vegetative growth, but mei4 mRNA does not accumulate even when heterochromatin is disrupted by deleting the sole H3K9 methyltransferase Clr4 (e.g., PMID: 22144463). In contrast, *mei4* mRNA accumulation can be readily observed in RNA degradation-defective *S. pombe* (e.g., PMID: 21317872). These findings indicate that post-transcriptional gene silencing is a dominant pathway for silencing genes embedded within heterochromatin, at least in some cases. By analogy, we hypothesize that although reduced H3K9me2 levels may permit transcription initiation, the resulting transcripts are rapidly degraded post-transcriptionally.

To investigate the potential post-transcriptional regulation of the mating-type genes, we examined their expression in fission yeast cells lacking Red1, a component of the MTREC complex involved in meiotic mRNA decay. As shown in Fig 3C, Red1 is dispensable for heterochromatin assembly. However, we observed increased mating-type gene expression in red1Δ (Fig 2C). This increase in *red1*∆ indicates that evident changes in mating-type gene expression can be observed when the RNA degradation pathway is inactivated, further supporting our hypothesis of post-transcriptional regulation. Consistent with this idea, we found that SJAG_05153, encoding the Mc-like mating-type transcription factor, contains a GTAAAC hexanucleotide motif, which is a signature of target RNAs degraded by an MTREC-dependent pathway in *S. pombe* (PMID: 22645662). We have now clarified this rationale in the manuscript.

*- Pab2 is proposed to form nuclear dots through phase separation based on their sensitivity to its own IDR and 1,6-hexanediol. While this might give hints for such biochemical behavior, it is not a direct proof. Unless further experiments are performed, the authors should tune down their conclusion in the manuscript.*

We thank Reviewer #3 for this important point. We acknowledge that our previous arguments for Pab2 phase separation based solely on IDR presence and 1,6-hexanediol sensitivity were premature. To directly address this concern, we performed an in vitro droplet formation assay. We expressed MBP-Pab2 (both WT and RA)-6×His tag fusion proteins (S4G Fig) in E. coli and purified them using Ni NTA beads followed by amylose resin. These purified proteins were then used in an in vitro assay after TEV cleavage (S4H Fig).

Our results demonstrate that Pab2^WT^ formed liquid-like droplets at a concentration of 2.5 µM, whereas a significantly higher concentration (25 µM) was required for Pab2^RA^ to form droplets (Fig 4F and S1 Movie). These droplets exhibited characteristic features of liquid condensates, including spherical shape and fusion events (S1 Movie). These findings provide strong evidence that Pab2 has an intrinsic capacity to undergo phase separation in vitro and support our idea that Pab2 nuclear foci are indeed biomolecular condensates. We have added a more detailed description of the in vitro assay and its results to the revised manuscript.

*- Yeast two hybrid data indicate that Pab2 interacts with Red5 and Rmn1 through its N-terminal domain (Fig S5A). It is a pity the authors did not characterize the pab2Nter∆ mutant as they did for pab2IDR∆ and pab2RRM∆. This could provide insights into the relevance of interactions (notably with Red5 and Rmn1), while possibly maintaining RNA-binding capacity.*

We thank Reviewer #3 for this excellent suggestion. We agree that characterizing the Pab2 N-terminal deletion mutant (Pab2ΔN) would provide valuable insights into the functional relevance of the interactions observed in the yeast two-hybrid assay (S5A Fig in the original manuscript, now shown in S6E Fig), particularly with Red5 and Rmn1, and potentially distinguish these functions from the RNA-binding activity of Pab2. As suggested, we generated a strain expressing Pab2ΔN-GFP from the endogenous pab2^+^ locus.

Our analysis revealed the following: (1) western blotting showed that the steady-state level of Pab2ΔN-GFP was comparable to that of Pab2-GFP (Fig S6F), indicating that deletion of the N-terminal domain does not affect protein stability; (2) Pab2ΔN cells exhibited sensitivity to cold and TBZ (S6H Fig), suggesting that the N-terminal domain is required for proper cellular function under stress conditions, (3) Pab2∆N-GFP localized to the nucleus but did not form nuclear condensates, though Red5 and Rmn1 still formed nuclear dots in Pab2∆N cells (S6G Fig); (4) similar to the pab2Δ strain, we observed a reduction in H3K9me2 at centromeres in Pab2ΔN cells (Fig 6F), indicating that the N-terminal domain is necessary for proper heterochromatin maintenance; (5) meiotic mRNAs (e.g., rec8 and spo5) accumulated in Pab2ΔN cells (Fig 6G), demonstrating that the N-terminal domain is also essential for meiotic mRNA decay, (6) RNA-IP showed that Pab2∆N significantly lost its ability to bind centromeric RNA (Fig 6H), and (7) Pab2 association with centromeres was reduced by deleting the N-terminal domain (Fig 6H).

These results indicate that the N-terminal domain of Pab2 is crucial for multiple functions, including heterochromatin maintenance and meiotic mRNA decay. The observed phenotypes strongly suggest that the N-terminal domain is essential for Pab2's roles in heterochromatin maintenance and meiotic mRNA decay in vivo. We have now incorporated these data and discussion into the revised manuscript.

*- Generally, ChIP and RNA-IP experiments lack proper controls, such as the use of untagged strains and/or the absence of relevant antibodies. The controls used for normalization should also be clearly written next to y axes or in figure legends.*

We thank Reviewer #3 for raising this important issue. To ensure the specificity of our ChIP and RIP assays, we have included data from untagged control strains in the relevant figures. The enrichment calculations, using the fba1^+^ locus for ChIP and act1 mRNA for RNA-IP as references, are indicated next to the y-axes in the relevant figures.

*- Details about the mass spectrometry procedure and analysis are absent.*

We thank Reviewer #3 for pointing out the lack of detail regarding our mass spectrometry procedures. We have now expanded the Materials and Methods section to provide a brief description of the mass spectrometry procedure together with the sample preparation method (see below). In addition to expanding the Materials and Methods section, we have deposited the raw mass spectrometry data and the detailed procedures to the PRIDE (Proteomics Identification Database): Project accession: PXD053432. This allows for full transparency and reproducibility of our results.

“Pab2 fused with the TurboID tag and its parental strains were lysed using RIPA buffer [50 mM Tris-HCl (pH 7.5), 150 mM NaCl, 1.5 mM MgCl_2_, 1 mM EGTA, 0.1% SDS, and 1% NP-40] supplemented with 0.4% sodium deoxycholate, 1 mM DTT, and 1 mM phenylmethylsulfonyl fluoride (PMSF). Biotinylated proteins in the cleared lysates were purified on streptavidin resin, digested with trypsin (Promega), and then subjected to desalting using a C18 StageTip. Liquid chromatography with tandem mass spectrometry (LC-MS/MS) was conducted with a Q-Exactive HF-X mass spectrometer linked to Easy 1200 nLC (Thermo Fisher Scientific).”

*- lines 175 to 177: Former work showed that Mmi1 contributes to pericentromeric silencing in S. pombe (PMID 28765164).*

We thank Reviewer #3 for this helpful comment. We acknowledge that our original text was unclear, leading to this confusion. The intended meaning was: “Previous studies reported that RNA degradation proteins, including Red1, Mmi1, and Erh1, were crucial for facultative, but not constitutive, heterochromatin assembly in S. pombe.” Indeed, the cited work (PMID 28765164) describes Mmi1's role in pericentromeric silencing, a downstream consequence of heterochromatin formation. As stated above, our focus is on heterochromatin assembly, and we have revised the text accordingly.

*- line 200: Ref 43 does not address Pab2 localization in S. pombe.*

We thank Reviewer #3 for their comment. We would like to clarify that Shishido et al. did examine Pab2 localization in S. pombe, as shown in their Fig. 1D and S1D Fig (please refer to: https://journals.plos.org/plosgenetics/article?id=10.1371/journal.pgen.1008598). These figures demonstrate that Pab2 colocalizes with Red1 and Mmi1, and Pab2 still forms nuclear foci in the absence of Red1. We apologize for any lack of clarity in our original text.
