# Supplementary material for: The nuclear poly(A)-binding protein Pab2/PABPN1 promotes heterochromatin assembly through the formation of Pab2 nuclear condensates
Source: PLoS Genet. 2025 Mar 31;21(3):e1011647. doi: 10.1371/journal.pgen.1011647 (PMC12002642; doi:10.1371/journal.pgen.1011647)
Supplement: Attachment — Submitted filename: 250305_Response to Reviewers.docx [file pgen.1011647.s024.docx]

**Our response to each point raised by the editor and reviewers.**

**Additional Editor Comments:**
*Please ensure that appropriate statistical tests for multiple comparisons are included in the experiments highlighted by Reviewer 3.*

We have addressed the Reviewer 3's concern regarding multiple comparisons (comment 8). Please see our detailed point-to-point responses described below.

*Additionally, I recommend clearly indicating the reference group (i.e., the baseline for statistical comparisons), as this is particularly important for evaluating additive or synergistic effects (for example, in Figure S3B for cid14∆ and cid14∆-RRM3A), and making changes to the text if needed..*

Thank you for your suggestion. Following Reviewer 3's suggestion, we have re-analyzed the statistical significance using one-way ANOVA. The re-analysis using one-way ANOVA did not reveal any reversals in statistical significance between analyzed groups, except for *spo5* expression in the RRM3A and telomeric transcripts in RRM∆ mutants. Consequently, we have revised all the relevant figures and their legends to clearly indicate the reference group (e.g., WT) and reflect the new statistical analysis. Necessary changes have also been made to the relevant text.

*Furthermore, regarding Reviewer 3’s comments on the role of leo1, you may wish to revise this section by either incorporating relevant details from your previous manuscript or removing it entirely for clarity.*

Thank you for the suggestion regarding Reviewer 3's comments. Following your advice, we have removed the sentence describing *leo1*∆. While this information was present in the previous manuscript, we determined that its inclusion in the current version would introduce unnecessary complexity, as it does not contribute directly to the main findings presented here.

**Reviewers' comments:**

Reviewer's Responses to Questions

*Reviewer #1: The authors correctly answered my comments. In addition, their responses to the other reviewers’ comments improve the manuscript. I believe the manuscript is now ready for publication in Plos Genetics.*

We are very pleased to hear that Reviewer #1 finds the manuscript now suitable for publication in PLOS Genetics. We greatly appreciate his/her positive assessment and continued support throughout the review process.

*Reviewer #2: The new submission has addressed all my concerns and has improved significantly. I am supportive for its publication on PLoS Genetics*

We are very pleased that Reviewer #2 finds the revised manuscript has addressed all his/her concerns and has improved significantly. We sincerely appreciate his/her support for publication in PLOS Genetics.

*Reviewer #3: Heterochromatin formation has been extensively studied, revealing many insights, yet numerous uncertainties remain. In this manuscript by Liu et al., the authors demonstrate that PABPN1 plays a key role in the formation of constitutive heterochromatin in the fission yeast S. japonicus. Deletion of the pab2 gene reduces the centromeric localization of the H3K9 methyltransferase and the chromatin remodeler, and impairs centromeric heterochromatin formation. This study demonstrates that S. japonicus, along with S. pombe, can serve as an excellent model system to study the mechanisms of heterochromatin formation.
The authors have adequately addressed the reviewers' comments from the first round of review; however, there are still some points that should be further improved, and certain parts lack detailed description, as described below.*

We appreciate your insightful feedback. We have responded to each point below:

*1_Has it been confirmed that the expression of genes such as mei4 and ssm4, which are referred to as meiotic genes, is truly meiosis-specific in S. japonicus? If not, please revise the wording in the text accordingly.*

We thank the reviewer for their insightful comment regarding the classification of mei4 and ssm4 as 'meiotic genes' in S. japonicus. We recognize that while these genes are known to be meiosis-specific (or meiosis-upregulated) in S. pombe, direct evidence confirming their strict meiosis-specificity in S. japonicus is lacking.

We agree that referring to them as 'meiotic genes' without explicit confirmation in S. japonicus is inaccurate. Therefore, we have revised the manuscript to refer to them as 'putative,' 'inferred,’ or 'potential' meiotic genes throughout the text. This change reflects the current state of our knowledge and avoids making unsubstantiated claims.

We appreciate the reviewer's attention to detail, which has allowed us to improve the accuracy and rigor of our manuscript.

*2_ While the ChIP-qPCR and RNA-IP data include information on normalization, there is no information provided on what the RT-qPCR data (Figure 1 and 2) were normalized to.*

We are sorry we did not include the description. We have revised the figure legends to clarify the normalization method used in our RT-qPCR data, **as shown below:**

**Fig 1B**

(B) RT-qPCR data from the four putative meiotic genes in WT and *pab2*Δ cells. The four putative meiotic mRNAs (mei4, ssm4, rec8, and spo5) were normalized to act1 mRNA to determine their relative expression levels. Data represent mean ± SD from three independent experiments.

**Fig 2B**

(B) RT-qPCR data from centromeric and telomeric transcripts in WT, *pab2*Δ, and *clr4-1* strains. Centromeric (*cen*) and telomeric transcripts (tel) were normalized to act1 mRNA to determine their relative expression levels. Data represent mean ± SD from three independent experiments.

**Fig 2C**

(C) RT-qPCR data from mating-type transcripts in WT, *pab2*Δ, *clr4-1*, and *red1*Δ strains. Transcripts from Mc-like and Pc-like genes (*Mc-like* and *Pc-like*) were normalized to act1 mRNA to determine their relative expression levels. Data represent mean ± SD from three independent experiments.

**Fig 6G**

(G) RT-qPCR analysis of transcripts derived from *mei4*^+^, *ssm4*^+^, *spo5*^+^, and *rec8*^+^, as well as centromeric (*cen*) and telomeric (*tel*) transcripts, in WT and *pab2*ΔN strains. The transcripts were normalized to *act1* mRNA to determine their relative expression levels. Data are presented as mean ± SD from three independent experiments.

**Fig 7D**

(D) RT-qPCR analysis of transcripts from *mei4*^+^, *ssm4*^+^, *spo5*^+^, and *rec8*^+^, as well as centromeric (*cen*) and telomeric (*tel*) transcripts, in WT and *pla1-3*. The transcripts were normalized to *act1* mRNA to determine their relative expression levels. Data are presented as mean ± SD from three independent experiments.

**S1G Fig**

(G) Putative meiotic gene expression in WT and *red1*Δ. RT-qPCR analysis of four inferred meiotic genes in WT and *red1*Δ cells. The four putative meiotic mRNAs (mei4, ssm4, rec8, and spo5) were normalized to act1 mRNA to determine their relative expression levels. Data are presented as mean ± SD from three independent experiments.

**S3A Fig**

(A) RT-qPCR analysis of centromeric and telomeric transcripts in WT, *cid14*Δ, *pab2*^RRM3A^, and *cid14*Δ-RRM3A strains. Centromeric (*cen*) and telomeric (*tel*) transcripts were normalized to act1 mRNA to determine their relative expression levels. Data are presented as mean ± SD from three independent experiments.

**S4F Fig**

(F) RT-qPCR analysis of transcripts from *mei4*^+^, *ssm4*^+^, *spo5*^+^, *rec8*^+^, as well as centromeric (*cen*) and telomeric (*tel*) transcripts, in Pab2-GFP and Pab2ΔIDR-GFP strains. The transcripts were normalized to *act1* mRNA to determine their relative expression levels. Data are presented as mean ± SD from three independent experiments.

**S4I Fig**

(I) RT-qPCR analysis of transcripts from *mei4*^+^, *ssm4*^+^, *spo5*^+^, *rec8*^+^, as well as centromeric (*cen*) and telomeric (*tel*) transcripts, in Pab2-GFP and Pab2^RA^-GFP strains. The transcripts were normalized to *act1* mRNA to determine their relative expression levels. Data are presented as mean ± SD from three independent experiments.

**S5A Fig**

(A) RT-qPCR analysis of transcripts from *mei4*^+^, *ssm4*^+^, *spo5*^+^, *rec8*^+^, as well as centromeric (*cen*) and telomeric (*tel*) transcripts, in WT, *pab2*ΔRRM, *pab2*^RRM3A^, and *pab2*^RRM3A-RA^ strains. The transcripts were normalized to *act1* mRNA to determine their relative expression levels. Data are presented as mean ± SD from three independent experiments.

**S6D Fig**

(D) RT-qPCR analysis of transcripts from *mei4*^+^, *ssm4*^+^, *spo5*^+^, *rec8*^+^, as well as centromeric (*cen*) and telomeric (*tel*) transcripts, in WT, *red5-1*, and *rmn1*Δ strains. The transcripts were normalized to *act1* mRNA to determine their relative expression levels. Data are presented as mean ± SD from three independent experiments.

**S8F Fig**

(F) RT-qPCR analysis of transcripts from *mei4*^+^, *ssm4*^+^, *spo5*^+^, *rec8*^+^, as well as centromeric (*cen*) and telomeric (*tel*) transcripts, in WT and *ppn1*^V354A, L355P^ strains. The transcripts were normalized to *act1* mRNA to determine their relative expression levels. Data are presented as mean ± SD from three independent experiments.

Also, we added “(target/*act1*)” to Figs 1B, 2B, 2C, 6G, 7D, S1G, S3A, S4F, S4I, S5A, S6D and S8F Fig. These changes will help the readers to understand how we normalized to calculate relative expression levels.

*3_Please add a brief explanation to the text about tbp1 and cdc2 in Figure 2D.*

We thank Reviewer #3 for raising this point. We acknowledge it is essential to explain what tbp1 and cdc2 are. We have revised the main text so that general readers can readily understand what they are, **as shown below:**

As shown in Fig 2D, Pab2 preferentially bound to RNAs derived from centromeres and meiotic mRNAs but did not preferentially associate with *tbp1* and *cdc2* mRNAs, which encode the TATA-binding protein Tbp1/TBP and the cyclin-dependent kinase Cdc2/Cdk1, respectively.

*4_Figure 3A. It would be helpful for general readers to have a more detailed explanation of the differences between H3K9me2 and me3.*

We appreciate the reviewer's suggestion to clarify the differences between H3K9me2 and H3K9me3 in Figure 3A for general readers. We have added the explanation to the main text, as shown below:

One of the hallmarks of heterochromatin is histone H3 Lys9 di- and tri-methylation (H3K9me2/H3K9me3), which provides binding sites for chromodomain proteins such as HP1 [4, 5]. While both H3K9me2 and H3K9me3 are repressive marks that are bound by chromodomain proteins, H3K9me3 is generally associated with a stronger and more stable form of transcriptional repression, often leading to heterochromatin formation, compared to H3K9me2 [45]. Since heterochromatic silencing is partially disrupted in *pab2*∆, we conducted chromatin immunoprecipitation followed by qPCR (ChIP-qPCR) to assess H3K9me levels in *pab2*Δ.

These changes will help the readers to understand the difference between di- and tri-methylation.

*5_Line506. The red5 knockout strain is not used; is it an essential gene?*

We appreciate the reviewer's inquiry regarding the red5 knockout strain. While we did not generate a *red5*∆ strain, we focused on isolating the red5-1 mutant due to previous evidence indicating the essentiality of red5^+^ in S. pombe. Given the high degree of evolutionary conservation between S. pombe and S. japonicus, we hypothesized that red5^+^ might also be essential in S. japonicus.

Furthermore, our experience has shown that genes essential in S. pombe are invariably essential in S. japonicus. Conversely, we have yet to identify a gene essential in S. pombe that is non-essential in S. japonicus. This empirical observation further supports our rationale for initially assuming the essentiality of red5^+^ in S. japonicus.

However, we acknowledge that the rationale for using the red5-1 mutant should have been more explicitly stated in the main text. We have revised the manuscript to include a clearer explanation of our experimental strategy and the reasoning behind our choice of the red5-1 mutant, as shown below:

To determine the roles of Red5 and Rmn1 in constitutive heterochromatin formation in S. japonicus, we generated a red5-1 mutant and an rmn1Δ strain. Given that Red5 is essential in *S. pombe*, we isolated the *red5-1* mutant in *S. japonicus* while avoiding potential lethality.

*6_Figure S7A, Lines 612-613. The figure does not clearly show an interaction between Rmn1 and Dri1.*

We appreciate the reviewer's comment regarding S7A Fig and the interpretation of the Rmn1-Dri1 interaction (Lines 612-613). We agree that the qualitative nature of the yeast two-hybrid assay, specifically the growth on selective plates, does not provide conclusive evidence for a strong or biologically significant interaction between Rmn1 and Dri1.

As the reviewer pointed out, while yeast cells carrying DBD-Rmn1 and AD-Dri1 showed growth on -Leu-Trp-His plates, suggesting a potential interaction, this growth was observed to be only slightly better than that of cells carrying DBD-p53 and AD-Dri1, which served as a proper negative control for the Y2H assay. Therefore, we acknowledge that any interaction between Rmn1 and Dri1, if it exists in vivo, is likely weak. We understand the need for more robust evidence and acknowledge that further quantitative analyses, beyond the scope of this current study, would be necessary to confirm the biological significance of this potential interaction.

To reflect this more accurately, we have revised Lines 612-613 as follows:

Yeast two-hybrid assays indicated slightly better growth of yeast cells carrying DBD-Rmn1 and AD-Dri1 on–Leu–Trp–His plates than those carrying DBD-p53 and AD-Dri1 (S7A Fig). This result suggests a possible, but weak, interaction between Rmn1 and Dri1. However, due to the qualitative nature of the assay, our observation does not provide sufficient evidence to confirm a direct and biologically relevant interaction between Rmn1 and Dri1. Further analyses are required to determine if there is any significant interaction. In addition, ChIP-qPCR analysis demonstrated that H3K9me2 levels at centromeres and telomeres in WT and *dri1*Δ were comparable (S7B Fig).

*7_Line846. There is no explanation of leo1 in the text, making it difficult to follow why leo1Δ is introduced.*

We thank Reviewer #3 for pointing out the lack of explanation for leo1Δ in the text (Line 846). We acknowledge that the mention of leo1Δ in the Discussion was an oversight.

In the original submission, we described our observation on *ppn1*∆*leo1*∆ and discussion regarding leo1Δ. However, based on feedback received during the initial review process, particularly regarding the interpretation of the leo1Δ data, we deleted all the descriptions on *leo1*∆ in the revised manuscript. Unfortunately, we inadvertently left a single mention of leo1Δ in the Discussion.

Considering the reviewer's comment and the editor's suggestion to streamline the manuscript, we have decided to remove the sentence containing leo1Δ. This eliminates the need for further explanation and prevents potential confusion.

We apologize for any inconvenience this may have caused.

*8_ Multiple t-tests are being conducted in the qPCR analyses (Figure 2C, 3A, 3B, 3C 4K, 5D, 5E, 5F, 6D, 6E, 6F, 6H, 7B, 8H, S2F, S3, S5A, S6D, and S7B), which is inappropriate. ANOVA and multiple comparisons should be performed instead.*

We thank the reviewer for pointing out the issue with the use of multiple t-tests in our qPCR analyses (Figure 2C, 3A, 3B, 3C 4K, 5D, 5E, 5F, 6D, 6E, 6F, 6H, 7B, 8H, S2F, S3, S5A, S6D, and S7B). We agree that one-way ANOVA with multiple comparisons is the more appropriate statistical method for these analyses.

In response to this comment, we have:

- Replaced all instances of *t*-tests in the aforementioned figures with ANOVA.
- Performed Dunnett's multiple comparisons to determine statistically significant differences between groups.
- Revised all relevant figure legends and text within the manuscript to accurately reflect the use of ANOVA and multiple comparisons.
- Updated the statistical method descriptions in the manuscript.

We believe these changes have strengthened the statistical rigor of our analyses and improved the overall quality of the manuscript. We appreciate the reviewer's careful review and constructive feedback.
